# The gut microbiota-mediated ferroptosis pathway: a key mechanism of ginsenoside Rd against metabolism-associated fatty liver disease

**DOI:** 10.1186/s13020-025-01121-1

**Published:** 2025-06-10

**Authors:** Wenjing Liu, Xian Zhou, Liyu Xiao, Xiaolan Huang, Dennis Chang, Xiaomei Zhong, Minjie Zeng, Yanfang Xian, Yanfang Zheng, Wei Huang, Rui Huang, Mingqing Huang

**Affiliations:** 1 The Affiliated People’s Hospital, College of Pharmacy, Fujian University of Traditional Chinese Medicine, Fuzhou, 350122 China; 2 NICM Health Research Institute, Western Sydney University, Westmead, NSW 2145 Australia; 3 Department of Pharmacy, Quan Zhou Woman’s and Children’s Hospital, Quanzhou, 362000 China; 4 Fuzhou Hospital of Traditional Chinese Medicine Affiliated to Fujian University of Traditional Chinese Medicine, Fuzhou, 350108 China; 5 School of Chinese Medicine, Faculty of Medicine, The Chinese University of Hong Kong, Shatin, N.T., Hong Kong SAR, China; 6 Department of Nursing, Mengchao Hepatobiliary Hospital of Fujian Medical University, Fuzhou, 350025 China

**Keywords:** Ginsenoside Rd, MAFLD, Gut microbiota, Lipid peroxidation, Ferroptosis, Nrf2

## Abstract

**Background:**

Ginsenoside Rd (G-Rd), found in *Panax* species, has shown therapeutic potential against metabolism-associated fatty liver disease (MAFLD), but its mechanism has not been well elucidated. This study investigated the key mechanisms of G-Rd in modulating the gut microbiome and lipid peroxidation-mediated ferroptosis pathway in MAFLD.

**Methods:**

A high-fat diet-induced MAFLD model was established. Ultrastructural changes in liver tissue were observed using transmission electron microscopy. Metagenomics were employed to detect alterations in gut microbiota and their metabolites. Biochemical analysis and immunohistochemistry were used to examine liver injury, blood lipids, lipid peroxidation-related indicators, and tissue iron content.

**Results:**

G-Rd significantly reduced liver injury and steatosis in MAFLD mice and downregulated the elevated relative abundance of *Firmicutes* and the Firmicutes/Bacteroidetes ratio. It also significantly reduced the abundances of *Faecalibaculum rodentium* while increasing *Muribaculum intestinale*, with its functional role being relevant to lipid metabolism regulation. Moreover, G-Rd ameliorated mitochondrial damage and inhibited the ferroptosis pathway in the liver, which was associated with antioxidant-related factors mediated by Nrf2 signaling. The liver protective effect of G-Rd was driven by the regulation of gut microbiota, as demonstrated by antibiotic cocktail treatment and fecal microbiota transplantation.

**Conclusions:**

G-Rd attenuated HFD-induced MAFLD by alleviating liver oxidative stress, lipid peroxidation, and ferroptosis through modulation of the gut microbiota. The antioxidant and anti-ferroptotic actions of G-Rd, mediated via the Nrf2 pathway, were found to contribute to the amelioration of liver injury and hepatic steatosis in MAFLD.

**Graphical Abstract:**

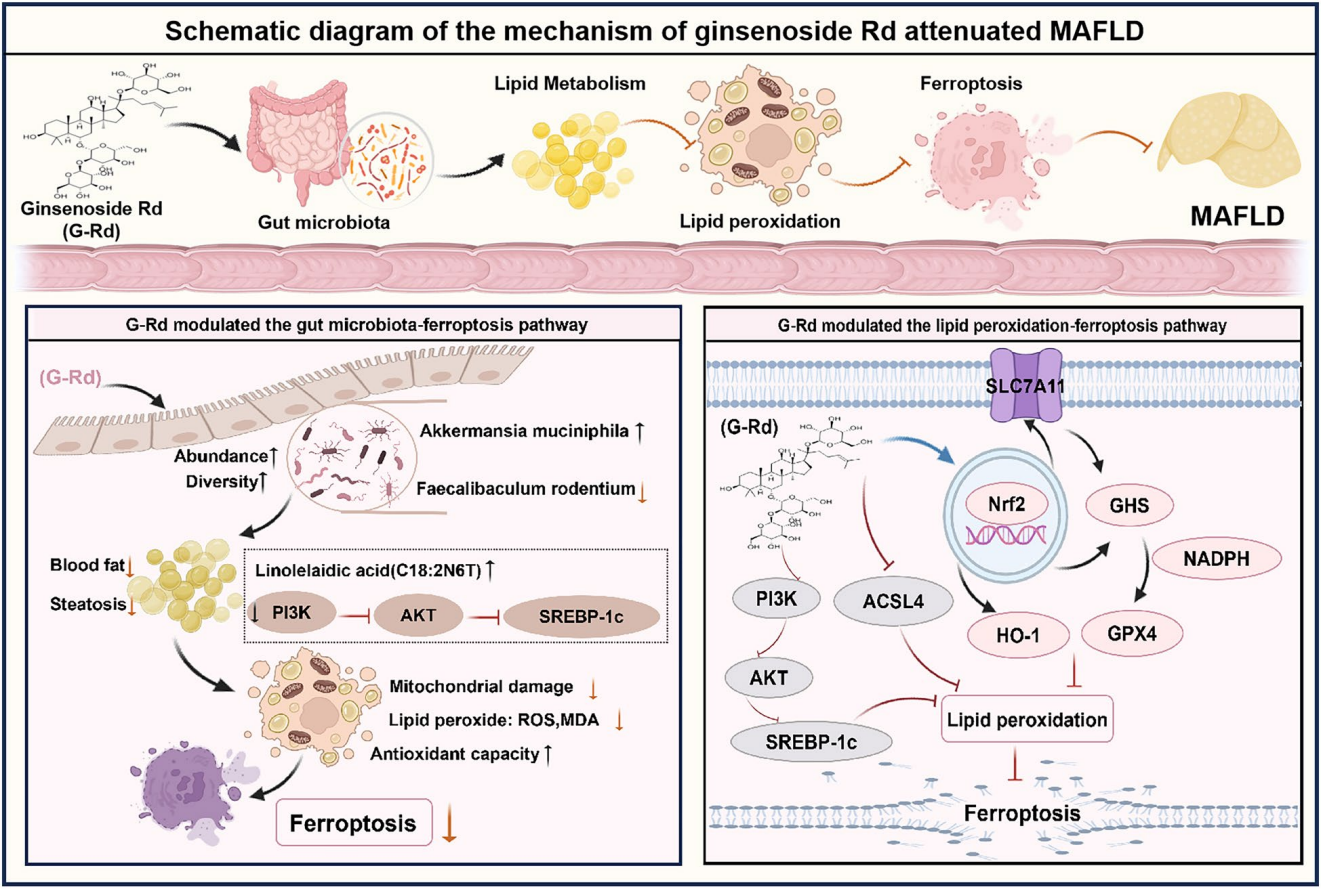

**Supplementary Information:**

The online version contains supplementary material available at 10.1186/s13020-025-01121-1.

## Introduction

Metabolic-associated fatty liver disease (MAFLD), which was renamed from non-alcoholic fatty liver disease (NAFLD), is a long-term liver condition strongly associated with diet, impacting around 32% of adults [1]. Its incidence is expected to rise steadily in the coming years [2]. MAFLD typically develops gradually, progressing to non-alcoholic steatohepatitis (NASH), liver fibrosis, and eventually resulting in liver cirrhosis or hepatocellular carcinoma [3]. As a metabolic disorder, MAFLD intricately interlaces with dietary habits, primarily characterized by intrahepatic lipid accumulation, inflammatory cascades, and oxidative stress [4, 5]. Notably, no viable pharmaceutical treatments are currently available for MAFLD. Lifestyle interventions, including weight management and exercise regimens, are currently the key therapeutic strategies for managing this condition [6, 7]. Therefore, potential novel pharmacotherapeutic interventions should be explored to mitigate the burgeoning burden of MAFLD.

The etiology of MAFLD remains enigmatic, yet emerging evidence underscores its multifaceted pathogenesis. Prior investigations have elucidated the pivotal role of physiological abnormalities, including obesity, insulin resistance and dyslipidemia [4, 5], in triggering and driving MAFLD progression. Notably, the symbiotic relationship between the gut microbiota and MAFLD has attracted considerable attention. The gut microbiota exerts profound influence over lipid metabolism by modulating lipid synthesis and transformation [8], thereby implicating its perturbation in the pathogenesis of MAFLD [9, 10]. Moreover, protracted exposure to a high-fat diet (HFD) precipitates dysbiosis within the gut microbiota, potentiating the risk of metabolic maladies, including MAFLD. Compellingly, investigations have substantiated the causal relationship between gut microbiota dysbiosis and MAFLD, as evidenced by targeted interventions such as probiotics, prebiotics, and fecal microbiota transplantation in clinic cohorts or animal models [11–13]. Mechanistically, dysregulated lipid metabolism precipitates lipid peroxidation, a process catalyzed by the oxidative degradation of fatty acids within organelles like mitochondria, acuminating in heightened reactive oxygen species (ROS) production [14]. Recent studies in lipid metabolism have revealed its regulatory role in ferroptosis, an iron-dependent form of programed cell death. For instance, ACSl4, a long-chain fatty acid acyl CoA synthase, catalyzes the biosynthesis of lipids rich in polyunsaturated fatty acids, thereby fostering the accumulation of lipid peroxidation intermediates and facilitating ferroptotic cell death [15].

Ferroptosis is characterized by the dysregulation of cellular iron homeostasis, culminating in the accumulation of redox-active iron, compromised antioxidant defenses, and heightened lipid peroxidation [15, 16]. Excessive intracellular iron levels precipitate a cascade of events, notably through the Fenton reaction, generating ROS and accentuating iron-induced cytotoxicity [17]. ROS-mediated assaults on cellular constituents, particularly the phospholipid bilayer, instigate lipid peroxidation, ultimately yielding malondialdehyde (MDA), a hallmark end-product of oxidative damage, thereby precipitating ferroptotic cell death [18]. Critical to the regulation of ferroptosis is the disruption of SLC7A11, an integral component of the cell’s system Xc, impairing glutathione (GSH) synthesis and attenuating glutathione peroxidase 4 (GPX4) activity, thus compromising cellular antioxidant defenses. Consequently, the ineffectiveness of GPX4 in metabolizing lipid peroxides permits unrestrained lipid oxidation by ferrous ion (Fe^2+^), fostering ROS generation and propagating the ferroptosis cascade [19–21]. In addition, ferroptosis can trigger inflammatory response of simple fatty liver degermation [22]. Notably, these mechanistic insights underscore the intricate interplay between ferroptosis and the pathogenesis of MAFLD. Exploration of the interplay between gut microbiota and lipid peroxidation offers a promising research strategy for MAFLD intervention and ferroptosis reversal. By modulating gut microbiota-mediated lipid peroxidation, therapeutic strategies may emerge to ameliorate MAFLD pathology and mitigate the progression of ferroptotic cell death.

Several Panax species, including *Panax ginseng* C. A. Mey., *Panax notoginseng* (Burk.) F. H. Chen, and *Panax quinquefolium* L., are traditionally used in Chinese medicine and have gained popularity in food and health products due to their tonic properties and high safety profile. Ginsenoside Rd (G-Rd), found in *Panax* species, possesses hepatoprotective properties, including MAFLD. Recently, it was found to lower ROS expression, lipid peroxides, and mitochondrial stress by activating Sirtuin 6 (SIRT6) in oleic acid and palmitic acid-induced mouse primary hepatocytes [23]. Furthermore, the mode of action of G-Rd was associated with inhibited ferroptosis in mice with carbon tetrachloride (CCl_4_)-induced acute liver injury through the cGAS/STING pathway [24]. However, the mechanism of G-Rd against MAFLD, particularly in relation to the regulation of gut microbiota and the lipid metabolism-mediated ferroptosis pathway, has not yet been reported. Therefore, this study aims to investigate the protective effect of G-Rd against MAFLD and to elucidate its molecular mechanism in alleviating MAFLD through the gut microbiota-lipid-metabolism-ferroptosis pathway.

## Materials and methods

### Animals and ethics statement

Male C57BL/6 mice (n = 78, 6–7 weeks old, 20 ± 2 g) were obtained from Beijing Huafukang Biotechnology Co. Ltd (License Number: SCXK-2019–0008). All animal procedures were approved by the Animal Care and Use Committee of the Fujian University of Traditional Chinese Medicine (Approval No.: FJTCM IACUC20221151). The mice were housed in specific pathogen-free conditions at 24 °C ± 1 °C, with a 12-h light/dark cycle and free access to water. The study followed the ARRIVE guidelines [25] and complied with the U.S. Public Health Service Policy on the Humane Care and Use of Laboratory Animals [26].

### Experimental design

#### Animal study 1

To assess the effect of G-Rd on a HFD-induced MAFLD mouse model, the following experimental procedures were conducted. After a one-week adaptation period, a total of 36 mice were randomly assigned to six groups (six mice per group). The control group was fed a standard diet, while the model group, fenofibrate (Fen, positive control) group, and three G-Rd treatment groups (low dose of 12.5 mg/kg, medium dose of 25 mg/kg, and high dose of 50 mg/kg) were provided with a HFD (Jiangsu Xietong Co., Ltd., China) consisting of 31.67% lard, 25.84% casein, 16.17% maltodextrin 10, 9.41% sucrose, 6.46% cellulose and mineral mix S10026B, 3.23% soybean oil, 0.39% L-cystine, 0.26% choline bitartrate and 0.13% vitamin mix V10001C for a duration of 12 weeks [27, 28]. Treatment with the respective drugs began in the ninth week. The model group received a daily oral gavage of 0.5% carboxymethyl cellulose (CMC)-Na, while the fenofibrate group (Fen) was administered a 5 mg/mL suspension of Fen (50 mg/kg, France liebofuni pharmaceutical company, batch no.: 32762) in 0.5% CMC-Na. For the G-Rd groups, mice were given daily oral gavages of G-Rd suspension in 0.5% CMC-Na at concentrations of 1.25 mg/mL (Rd 12.5), 2.5 mg/mL (Rd 25), and 5 mg/mL (Rd 50) at a dosage of 0.1 mL per 10 g body weight for four weeks [23, 24]. G-Rd was obtained from Shanghai Yuanye Bio-Technology Co., Ltd (purity > 98%, batch no.: J11HS184464). Please refer to supplementary material 1 for the dosing schedule of the animal study 1.

#### Animal study 2

Antibiotic-induced gut microbiome disruption was used to evaluate the effects of G-Rd treatment. After a week of adaptive feeding, C57BL/6 mice (n = 24) were randomly divided into four groups: the control group with a regular diet (Con), the antibiotic + HFD group (Mod-Ab), the antibiotic + HFD + G-Rd group (Rd-Ab), and the HFD + G-Rd 25 mg/kg group (Rd), with n = 6 per group. A pseudo-sterile mouse model was established in the Mod-Ab and Rd-Ab groups using a four-drug antibiotic cocktail, which included vancomycin hydrochloride (McLin, V871983, China) 500 mg/L, ampicillin sodium (McLin, A830931, China) 1 g/L, neomycin sulfate (McLin, N6063, China) 1 g/L, and metronidazole (McLin, M813526, China) 1 g/L, all mixed into the daily drinking water. This was followed by the administration of the corresponding treatments. After 4 weeks of antibiotics (AVNM) treatment, the Rd-Ab and Rd groups were given 25 mg/kg of G-Rd by gavage, while the Con and Mod-Ab groups received an equivalent volume of 0.5% CMC-Na daily by gavage for 8 weeks. The entire experimental period lasted 12 weeks.

#### Animal study 3

Fecal microbiota transplantation (FMT) was utilized to explore the involvement of gut microbiota in the liver-protective effects of G-Rd. A total of 18 C57BL/6 mice were randomly assigned to three groups: the control group, the model group, and the G-Rd 2.5 mg/mL (Rd 25) group, with six mice per group. After one week of acclimatization, the control group was fed a regular diet, while the other groups were given an HFD as described earlier. Subsequently, all groups received an antibiotics cocktail (0.2 g/L ampicillin, 0.2 g/L neomycin, 0.2 g/L metronidazole, and 0.1 g/L vancomycin) daily for two weeks to deplete their gut microbiota [29]. After the two-week treatment, mice from the control, model, and Rd 25 groups from Study 1 were orally administered 200 µL of fecal suspension twice a week for four weeks, while they were provided with sterilized drinking water. At the start of the seventh week, the control group resumed a regular diet, while the other groups continued with the HFD for an additional six weeks. For the flowchart of Animal Study 2, please refer to Supplementary Material 1.

At the conclusion of the 12-week treatment period, the mice were fasted for 12 h and then anaesthetised via abdominal injection of 1.1 g/kg of 25% urethane. Blood samples were collected through eyeball removal and centrifuged at 3500 rpm at 4 °C for 10 min to obtain serum for further analysis. Liver weights were also recorded. A portion of the liver tissues was fixed in paraformaldehyde for hematoxylin and eosin (H&E) staining, while the remaining tissue was promptly frozen at − 80 °C for subsequent analysis.

### Measurements of body weight, random blood glucose and Lee’s index

Two hours after the final treatment, body weight and random blood glucose (RBG) levels were measured. For the RBG measurement, blood was collected from the tail and tested using a glucometer (Roche, Indianapolis, United States) with blood glucose test strips. Lee’s index (%) was calculated using the following formula: Lee’s index (%) = [weight ^ (1/3) (g) × 1000]/body length (cm) × 100%.

### Measurement of serum transaminase enzymes and blood lipids

Blood samples were obtained through eyeball extraction and left at room temperature for 60 min to allow complete coagulation. Afterward, the samples were centrifuged at 3500 rpm for 10 min at 4 °C. The serum supernatant was separated and used to measure levels of aspartate transaminase (AST), alanine transaminase (ALT), triglycerides (TG), total cholesterol (TC), non-esterified fatty acids (NEFA), and low-density lipoprotein cholesterol (LDL-C) using commercial kits from Nanjing Jiancheng Bioengineering Institute, Nanjing, China.

### Measurement of serum oxidative stress, blood and liver lipids and tissue iron content biomarkers in liver

Liver tissues from each mouse were homogenised in phosphate buffered saline (PBS) and then centrifuged at 2500 g for 10 min. The levels of iron (Fe) content and ROS were measured using commercial kits from Nanjing Jiancheng Bioengineering Institute, Nanjing, China. Additionally, serum oxidative stress biomarkers and blood lipid biomarkers in liver samples, including superoxide dismutase (SOD), glutathione peroxidase (GSH), malondialdehyde (MDA), triglycerides (TG) and total cholesterol (TC), were also assessed using kits from the same supplier.

### Gut microbiota sequencing and data analysis

At the conclusion of the 12-week treatment period, fecal samples from the control, model and Rd 25 groups were collected in 1.5 mL centrifuge tubes on ice prior to postmortem examination. These samples were then sent to Wekemo Tech Group Co., Ltd., Shenzhen, China, for gut metagenomic sequencing and data analysis. DNA extraction followed the method outlined in a previous study [30], with purity and concentration verified using agarose gel electrophoresis. Metagenomic sequencing was performed on the Illumina Novaseq platform (USA) with paired-end 150 bp reads and an insert size of 350 bp per sample. The raw reads were processed to eliminate low-quality and ambiguous bases using the following trimmomatic parameters: illuminaclip: adapter path: 2:30:10, sliding window: 4:20, minlen:50. Reads aligning to the mouse genome reference and host DNA were removed using Bowtie 2 with ‘very sensitive’ settings. Species diversity and composition were analysed and classified using the Kraken2 (2018) program. Abundance estimation was conducted using Kraken (Bracken), a Bayesian method to determine the DNA sequence abundance in each sample. The study also analysed the percentage of sequences from each sample across the entire dataset, from kingdom to species level. Principal coordinate analysis (PCoA) was employed to examine species composition differences among the groups. Clustering analysis was used to assess the similarity of species composition across samples. Linear discriminant analysis (LDA) effect size (LEfSe) was used to identify species with high abundance in each group. Significant and enriched bacteria in each group were identified using LEfSe, and their metabolite pathways were analysed through the Kyoto Encyclopedia of Genes and Genomes (KEGG). A Circos plot was created to visualize the top 10 KEGG pathways in each sample.

### Metabolomic analysis

For each sample, 100 mg of mashed feces was mixed with 500 μL of an 80% methanol aqueous solution, vortexed, and placed on ice for 5 min. The mixture was then centrifuged at 15,000 rpm and 4 °C for 20 min. The supernatant was diluted with mass spectrometry-grade water to achieve a methanol concentration of 53%, then centrifuged again to collect the supernatant for ultra-high performance liquid chromatography-tandem mass spectrometry (UHPLC-MS/MS) analysis.

UHPLC-MS/MS analyses were conducted using a Vanquish UHPLC system (ThermoFisher, Germany) coupled with an Orbitrap Q Exactive^TM^HF mass spectrometer (Thermo Fisher, Germany). Samples were injected onto a Hypersil Gold column (100 × 2.1 mm, 1.9 μm) and analysed with a 17-min linear gradient at a flow rate of 0.2 mL/min. In the positive polarity mode, eluent A was 0.1% formic acid (FA) in water, and eluent B was methanol. In the negative polarity mode, eluent A was 5 mM ammonium acetate at pH 9.0, and eluent B was methanol. The solvent gradient was as follows: 2% B for 1.5 min; 2–85% B for 3 min; 85–100% B for 10 min; 100–2% B for 10.1 min; and finally 2% B for 12 min. The Q Exactive™ HF mass spectrometer was operated in both positive and negative polarity modes with a spray voltage of 3.5 kV, a capillary temperature of 320 °C, a sheath gas flow rate of 35 psi, an auxiliary gas flow rate of 10 L/min, an S-lens RF level of 60 and auxiliary gas heater temperature of 350 °C.

Data was processed using the CD3.1 library search software, where metabolites were first screened based on retention time, mass-to-charge ratio, and other parameters (retention time deviation of 0.2 min, mass deviation 5 ppm). For more precise identification, peak extraction was performed (with a mass deviation of 5 ppm, a signal intensity deviation of 30%, and a signal-to-noise ratio of 3), followed by simultaneous peak area quantification. After integrating target ions, molecular ion peaks and fragment ions were predicted using the molecular formula and compared with mzCloud (www.mzcloud.org/). Additionally, comparisons were made with mzVault and MassList databases, and background ions were removed using blank samples. Following normalization, relative peak areas were calculated. Compounds with a coefficient of variation (CV) in relative peak area greater than 30% were excluded. The identified metabolites were annotated using the KEGG (www.genome.jp/kegg/pathway.html) and LipidMAPS databases (http://www.lipidmaps.org/).

### Histopathological staining

H&E staining (H&E): The pathological staining procedure followed the methods described in our previous study [31]. Liver tissue from mice was collected, fixed in 4% paraformaldehyde, and embedded in paraffin. The paraffin blocks were sectioned into 4 μm slices, deparaffinized, washed with water, and stained using an H&E staining kit (G1120, Solarbio) according to the manufacturer’s instructions. The stained sections were used to assess the MAFLD activity score (NAS) as previously outlined [32].

Oil Red O staining (ORO): Frozen liver tissue sections, 6 µm thick, were stained using the Oil Red O kit (R20642, Shanghai Bio-Technology Co., Ltd, Shanghai, China). The stained tissues were observed under a light microscope.

Perl’s Iron staining: Paraffin-embedded liver tissue was sectioned into 4 μm thick slices, deparaffinized, washed in water, and stained with the Prussian blue iron stain kit (with nuclear fast red) (G1422, Solarbio) following the manufacturer’s instructions. The stained tissues were analysed under a light microscope, and the images were processed using Image Pro Plus 6.0 software.

Ultrastructure observation by transmission electron microscopy (TEM): The liver sample was fixed in 2.5% glutaraldehyde (pH 7.4) for 2 h, washed three times with 0.1 M phosphate buffer (pH 7.2), and further fixed in 1% osmium tetroxide at 4 °C for 2 h. Following fixation, the sample was dehydrated through a series of ethanol solutions and embedded in Epon-Araldite resin. The embedded sample was polymerized, and semi-thin sections were prepared before ultrathin sections were cut for microscopic analysis. The sections were stained with 3% uranyl acetate and 2.7% lead citrate before being observed using an HT7800 transmission electron microscope.

### Western blot analysis

Liver tissue was homogenized, and radioimmunoprecipitation (RIPA) lysis buffer containing 1% protease/phosphatase inhibitor was added. The mixture was then centrifuged at 12,000 rpm at 4 °C for 10 min, and the supernatant was collected. Protein concentration was measured using the BCA Protein Assay Kit (Beyotime Biotechnology, China). Total protein from each sample (6 μg/μL) was combined with 5X protein loading buffer (Beyotime Biotechnology, China) and heated at 100 °C for 10 min to denature the proteins. The proteins were separated via SDS-PAGE electrophoresis and transferred to a PVDF membrane. The membranes were then incubated with 5% skim milk in TBS-T (1X tris-buffered saline and 0.1% Tween 20) for 1.5 h at room temperature. Following this, the membranes were incubated overnight at 4 °C with the primary antibodies listed in Table 1. On the following day, the membranes were washed three times with TBS-T and incubated with the secondary antibody (HRP Goat Anti-mouse IgG H + L or HRP Goat Anti-rabbit IgG H + L, 1:5000) for 1.5 h. Immunoreactive bands were visualized using the ChemiDoc XRS + (BioRad, Hercules, USA), and the band intensities were analyzed using Image Lab software.

**Table 1:** Antibodies used in this study

Antibodies	Supplier (Cat. No.)	Host	Dilution
AKT	Cell Signaling Technologies (4691S)	Rabbit	1:1000
p-AKT	Cell Signaling Technologies (4060S)	Rabbit	1:1000
PI3K	Abcam (ab154598)	Rabbit	1:1000
p-PI3K	Abcam (ab278545)	Rabbit	1:1000
SREBP-1c	SANTA CRUZ (sc-13551)	Mouse	1:500
TNF-α	Abcam (ab109322)	Rabbit	1:1000
NLRP3	Cell Signaling Technologies (15101S)	Rabbit	1:1000
ASC	Cell Signaling Technologies (67824S)	Rabbit	1:1000
caspase-1	Cell Signaling Technologies (89332S)	Rabbit	1:500
COX-2	Proteintech (12375-1-AP)	Rabbit	1:1000
IL-18	Abcom (ab191860)	Rabbit	1:1000
IL-1β	Cell Signaling Technologies (63124S)	Rabbit	1:1000
SLC7A11	Proteintech (26864-1-AP)	Rabbit	1:1000
GPX4	Cell Signaling Technologies (59735S)	Rabbit	1:1000
ACSL4	Proteintech (22401-1-AP)	Rabbit	1:5000
NADPH	Abcam (ab133303)	Rabbit	1:1000
Keap1	Cell Signaling Technologies (8047S)	Rabbit	1:500
Nrf2	Cell Signaling Technologies (33649S)	Rabbit	1:1000
HO-1	Cell Signaling Technologies (82206S)	Rabbit	1:1000
NQO1	Abcam (ab80588)	Rabbit	1:1000
GCLC	Proteintech (12601-1-AP)	Rabbit	1:1000
β-actin	Proteintech (SA00001-1)	Mouse	1:5000

#### Statistical* analysis*

Data from all experiments were analyzed using GraphPad Prism 8.0 software and are presented as mean ± standard error of the mean (n ≥ 3). Group comparisons were performed using one-way Analysis of Variance (ANOVA) or non-parametric tests, with statistical significance set at *p* < 0.05. Pearson correlation analysis was also conducted using GraphPad Prism.

## Results

### G-Rd mitigated hepatic steatosis and liver injury in MAFLD mice

#### G-Rd reduced body weight, Lee’s index and RBG level

**Fig. 1:**
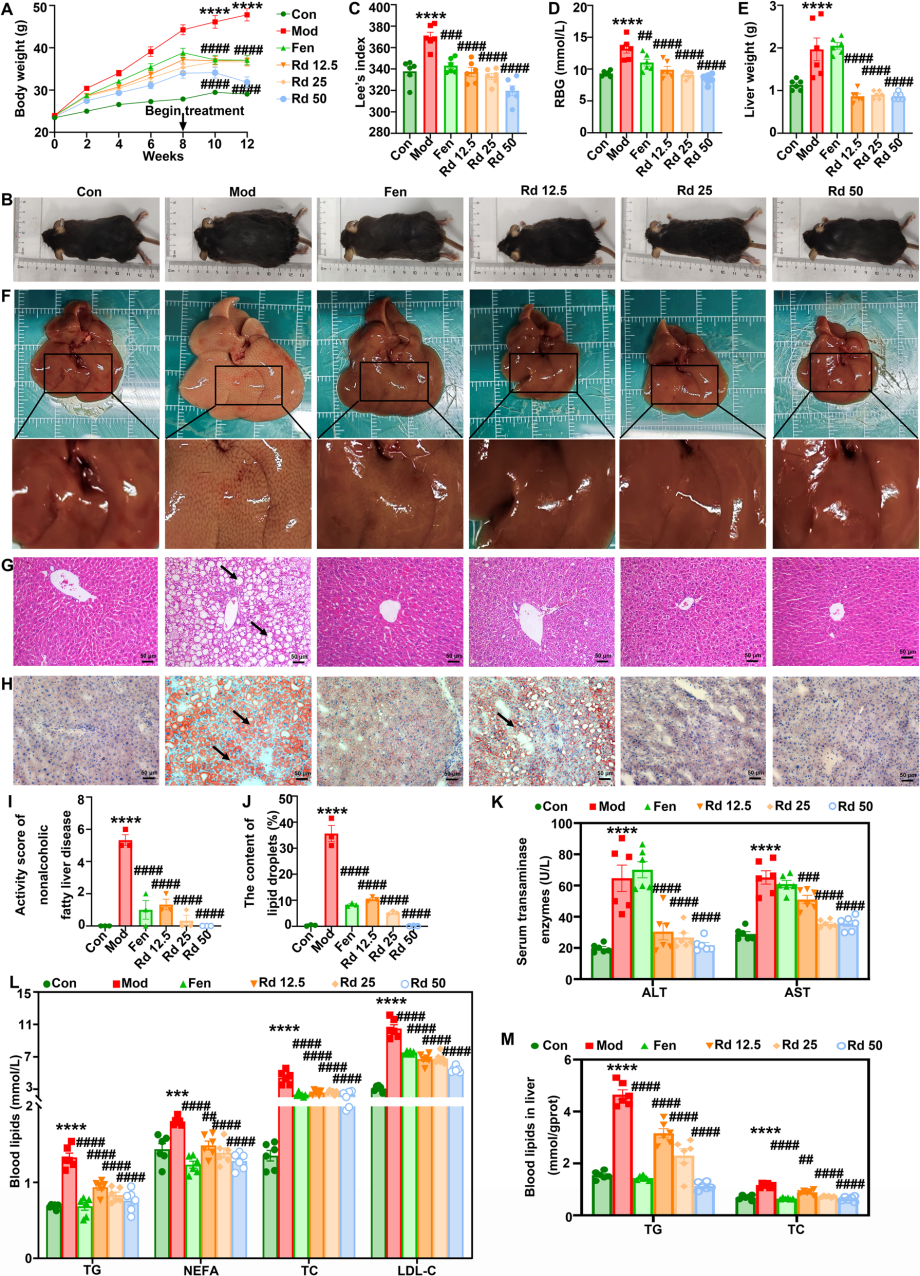
G-Rd attenuated liver injury and hepatic lipid accumulation in MAFLD mice (n = 6 per group, ≥ 3 individual experiments each sample). G-Rd (12.5, 25 and 50 mg/kg) significantly reduced (**A**) body weight (g), (**B**) body size, (**C**) Lee’s index (%), (**D**) RBG levels (mmol/L), and (**E**) liver weight (g). Representative (**F**) images of hepatic morphology, (**G**) H&E staining (scale bar = 50 μm), and (**H**) Oil Red O staining (scale bar = 100 μm). (**I**) The activity score of MAFLD: total score ≥ 5 = NASH, < 3 = non-NASH, 3–4 = limit to NASH. **J** Positive rate of Oil Red O staining (%), representing the lipid droplet content. Serum levels of (**K**) ALT, AST (U/L), (**L**) TG, NEFA, TC and LDL-C and (**M**) levels of TG, TC in liver. TC ***p < 0.001, ****p < 0.0001 *vs.* Con; ^##^*p* < 0.01, ^###^*p* < 0.001, *vs.* Mod group. Con: Control group, Mod: Model group, Fen: Fenofibrate group, Rd 12.5: 12.5 mg/kg ginsenoside Rd group, Rd 25: 25 mg/kg ginsenoside Rd group, Rd 50: 50 mg/kg ginsenoside Rd group

The effects of G-Rd were examined on body weight, Lee’s index and RBG level in MAFLD mice. From week 0 to week 12, the model group consistently showed higher body weight compared to the control group, while the body weight in the G-Rd groups was significantly lower than that of the model group (Fig. 1A-B). A similar trend was observed in Fen group in lowering body weight. In addition, G-Rd at all dosage levels significantly reduced the elevated Lee’s index, RBG levels, and liver weight (Fig. 1C-E) compared to the model group. The results demonstrated the dose-dependent effect of G-Rd in improving glycemic control and reducing liver fat content. Oral administration of Fen also reduced these markers.

#### G-Rd attenuated pathological and morphological changes of liver

The effect of G-Rd on liver morphology and histology in MAFLD mice was examined (Fig. 1F). The liver tissue surface in the control group appeared smooth. In the model group, the liver was enlarged with discernible white adipose particles on the surface. In the G-Rd groups, the liver volume was reduced, the surface was smooth and ruddy, and the morphology and colour tended to return to normal. In the Fen group, the degree of liver enlargement remained unchanged, however, no discernible white fat particles were observed on the surface.

Histopathological staining of liver tissues was conducted to assess the histopathological changes with and without G-Rd treatment in MAFLD mice. The control group exhibited normal liver tissue histology (Fig. 1G), which was also observed in the Fen group. In contrast, the model group showed clear signs of lipid droplet accumulation and ballooning, which are key pathological features of NASH. In the G-Rd groups, however, such pathological changes were less pronounced. In particular, G-Rd at 50 mg/kg displayed a morphology compared to that of the control group. Statistical analysis demonstrated that the G-Rd groups had significantly lower MAFLD liver injury score (*p* < 0.0001) than that of the model group (Fig. 1I). A similar trend was observed in the Oil Red O staining for lipid droplet detection (Fig. 1H, J). The liver tissues in the model group showed a large area of red hepatocytes and fat droplets compared to the control group (*p* < 0.0001), suggesting significant lipid accumulation. G-Rd dose-dependently reduced the percentage of lipid droplets in comparison to the model group, as did the Fen group (all *p* values < 0.0001).

#### G-Rd lowered serum transaminase enzymes and blood lipids

G-Rd significantly reduced AST and ALT levels in a dose-dependent manner compared to the model group (Fig. 1K), indicating its potential to alleviate hepatic injury in MAFLD mice. Notably, no significant changes in AST and ALT levels were observed in the Fen group. Furthermore, G-Rd treatment notably lowered levels of TG, NEFA, TC, and LDL-C in the blood compared to both the model and Fen groups (*p* < 0.0001), where these parameters were found to be excessively elevated (Fig. 1L). In addition, G-Rd treatment significantly lowered levels of TG and TC in the liver compared to the model (*p* < 0.0001). Overall, G-Rd effectively reduced hepatic steatosis and liver damage in the MAFLD mice.

### G-Rd regulated the gut microbiota and metabolites in MAFLD mice

#### The effect of G-Rd in modulating the abundance and diversity of gut microbiota

**Fig. 2:**
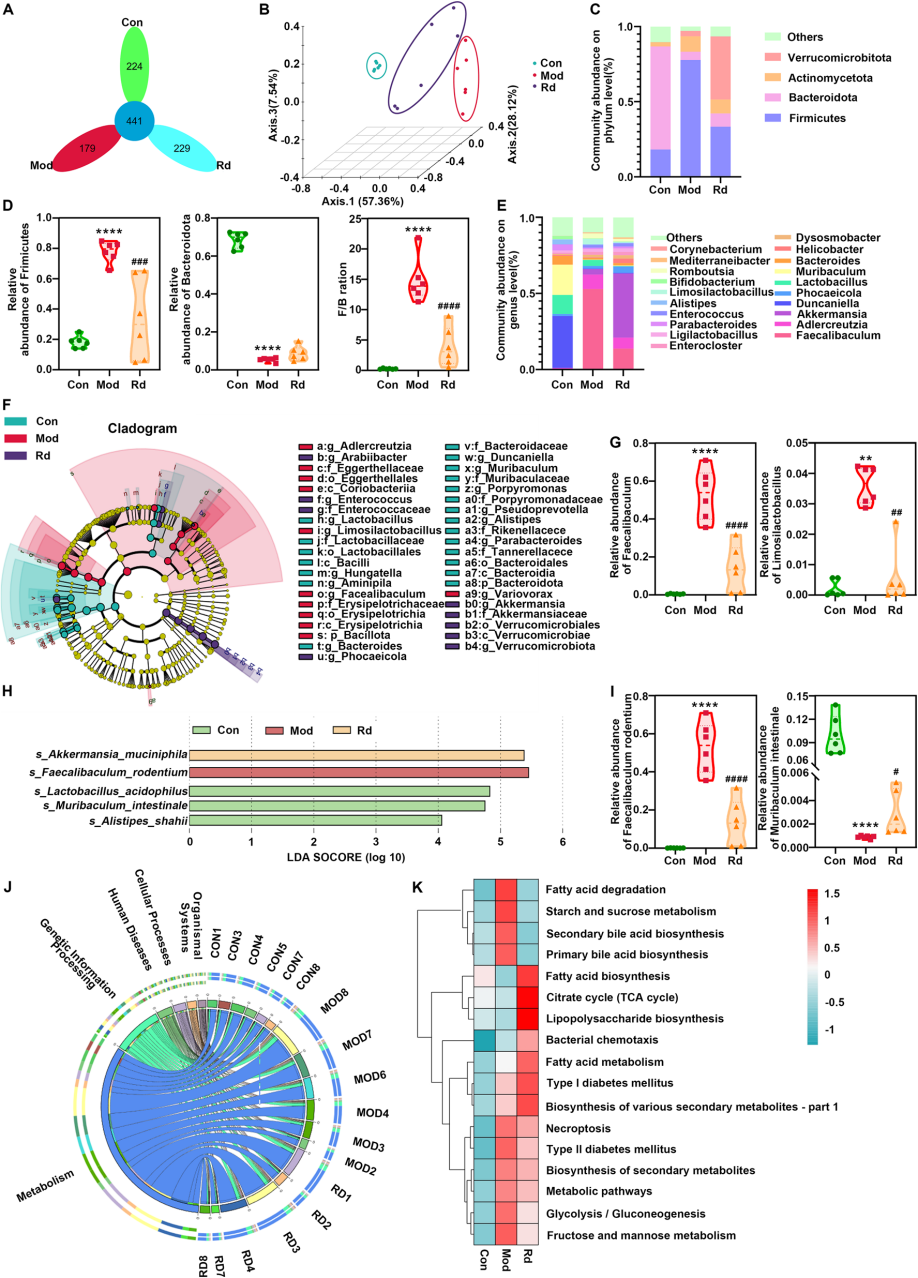
G-Rd restored the impaired diversity and modulated abundances of the gut microbiome in MAFLD mice (n = 6 in each group). **A** OTUs analyse for the diversity of gut microbiome in each group. **B** The β-diversity analysed by PCoA. **C** The relative abundance of the four top-ranked phyla among the three groups. **D** The relative abundance of Firmicutes and Bacteroidetes and the F/T ratio. **E** The relative abundance of the twenty top-ranked genera among the three groups. **F** Feature genera in the three groups at the LDA4 level analysed by LDA effect size. **G** The relative abundance of Faecalibaculum and Limosilactobacillus. **H** Feature phyla in the CON, MOD, and G-Rd groups at the LDA4 level analysed by LEfSe. **I** The relative abundance of *Faecalibaculum rodentium* and *Muribaculum intestinale*. **J** Altered KEGG pathways (level 1) among the three groups as shown in the Circos plots. **K** Heat map of the altered KEGG pathways. ****p < 0.0001 *vs.* Con; ^#^*p* < 0.05, ^##^*p* < 0.01, ^###^*p* < 0.001 *vs.* Mod group. Con: Control group, Mod: Model group, Rd: 25 mg/kg ginsenoside Rd group

Metagenomic shotgun sequencing was performed to understand the mechanisms of action of G-Rd in attenuating MAFLD, particularly in relation to the regulation of gut microbiota. Since Fen did not show any significant effect in attenuating liver injury, the group was excluded from the following assays. As shown in Fig. 2A, the unique features in the model group (179) were lower than those in the control group (224), suggesting impaired abundance and diversity of the gut microbiota in MAFLD. However, the unique features were restored in the G-Rd group (229), which were much higher than those in the model group and comparable to the control group.

The β-diversity analysis using PCoA (Fig. 2B) indicates that the sample distribution in the model group differed from that in the control group, with principal component 1 accounting for 57.36%, principal component 2 for 28.12%, and principal component 3 for 7.54%.

#### The effect of G-Rd in modulating the abundance of gut metagenome at the phylum level

We have compared the abundance of dominant phyla in the three groups. The top four dominant phyla were *Firmicutes, Bacteroidota, Verrucomicrobiota and Actinomycetota*, which together accounted for over 90% of the total abundance (Fig. 2C). The relative abundance of *Firmicutes* and the *Firmicutes/Bacteroidota* (F/B) ratio in the model group were significantly higher than those in the control group (*p* < 0.0001, Fig. 2D). The relative abundance of Bacteroidetes in the model group was significantly decreased (*p* < 0.0001). Notably, G-Rd reduced the relative abundance of Firmicutes and the F/B ratio (*p* < 0.001, *p* < 0.0001), although no significant effect was observed for Bacteroidota.

#### The effect of G-Rd in modulating abundance of gut metagenome at the genus level

The top 20 dominant genera among the three groups include *Faecalibaculum, Adlercreutzia, Akkermansia, Muribaculum, Bacteroides*, which together constitute over 85% of the overall detected abundance (Fig. 2E). LEfSe analysis suggested distinct feature genera among the three groups. Notably, the high abundance of *Faecalibaculum* in the model group was much lower in the G-Rd group (Fig. 2F).

Specific statistical comparisons were conducted to assess the changes in the relative abundance of *Faecalibaculum* and *Limosilactobacillus*. Their increased relative abundances in the model group (*p* < 0.0001 and *p* < 0.01 vs. the control group) were significantly reduced in the G-Rd group (Fig. 2G), suggesting the regulatory ability of G-Rd for these genera.

#### The effect of G-Rd in modulating abundance of gut metagenome at the species level

Based on the results obtained at the phylum and genus level (Fig. 2H), LEfSe analysis suggested that feature species in the control group at the LDA4 level included *Muribaculum intestinale*, *Lactobacillus acidophilus*, and *Alistipes shahii*; these were distinct from those in the model group, which was enriched with *Faecalibaculum rodentium*. Interestingly, *Akkermansia muciniphila* was the only predominant species in the G-Rd group, suggesting its high expression in this group. Statistical analysis revealed that the elevated *Faecalibaculum rodentium* level in the model group (*p* < 0.0001 vs. the control group) was reduced in the G-Rd group (*p* < 0.0001 vs. model group), which corresponded to the change in *Faecalibaculum* at the genus level (Fig. 2I). Additionally, *Muribaculum intestinale* was significantly restored in the G-Rd group (*p* < 0.05) compared with the model group.

### G-Rd regulated the functional components of metagenome in MAFLD mice

The Circos plots revealed that G-Rd most significantly regulated metabolism-related functions among the functional components at level 1 (Fig. 2J). A detailed comparison of the modulated KEGG pathways between the three groups is shown in the heat map (Fig. 2K). Fatty acid degradation and biosynthesis exhibited clear differences between the control and model groups, while the G-Rd group primarily displayed pathway patterns similar to those of the control group.

### G-Rd regulated lipid metabolism in MAFLD mice

Based on the previous results, which suggest that the predominant effect of G-Rd is regulated through lipid metabolism pathways, the levels of key metabolites in the fecal samples were examined as part of the lipid metabolism in the three groups.

**Fig. 3:**
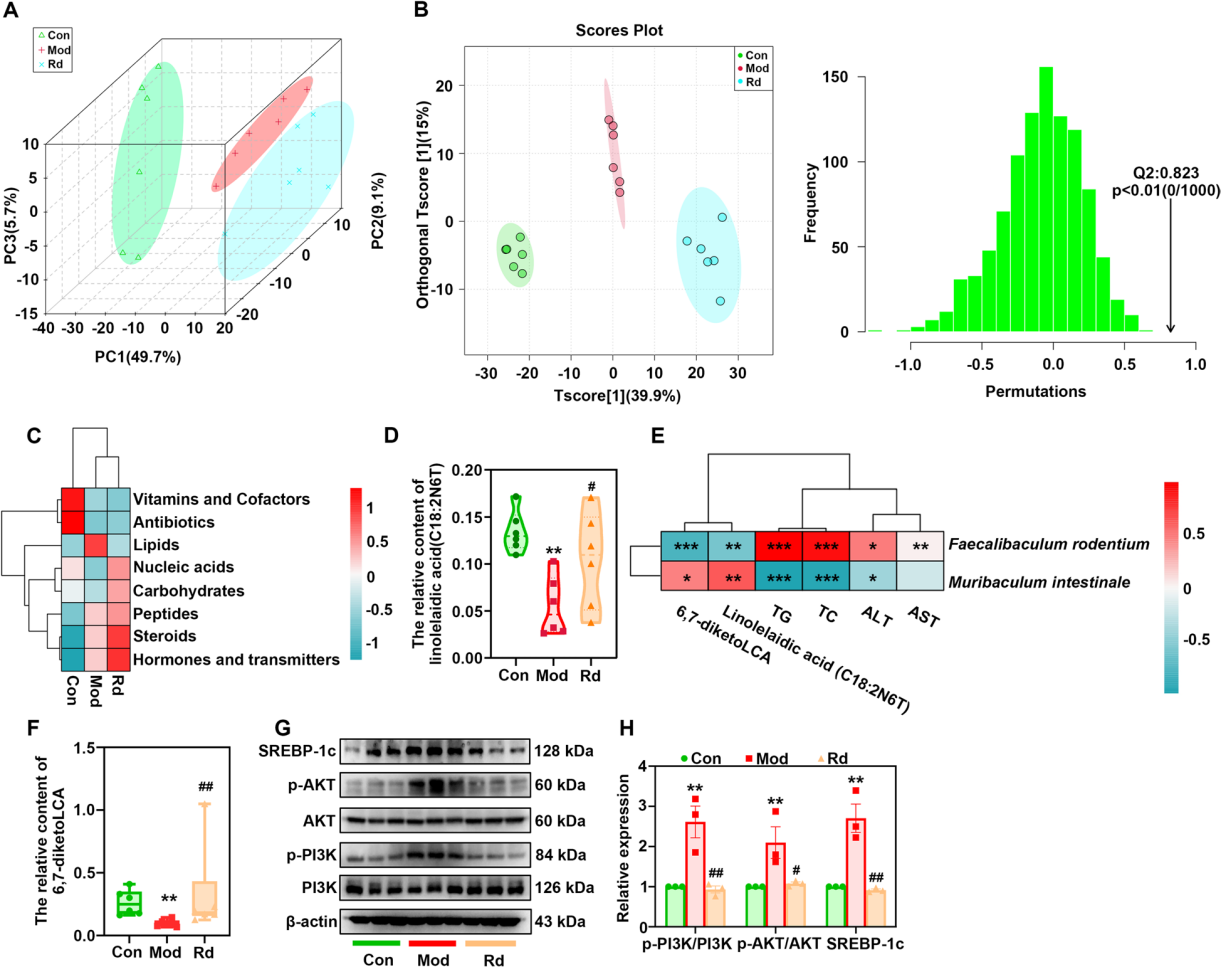
G-Rd restored lipid metabolism in MAFLD mice (n = 6 in each group). **A** The β-diversity analysed by PCoA. **B** The orthogonal partial least squares discriminant analysis (OPLS-DA) among the three group. **C** Heatmap analysis of metabolites. **D** The relative content of linolelaidic acid (C18:2N6T). **E** The correlation analysis of gut microbiota, metabolites and pharmacodynamics indexes of MAFLD. **F** The relative content of 6,7-diketoLCA. **G**-**H** Immunoblotting analysis of relative protein expressions of PI3K, p-PI3K, AKT, p-AKT, SREBP-1c to β-actin.

Principal component analysis (PCA) and orthogonal partial least squares discriminant analysis (OPLS-DA) suggested that the distribution of the total metabolites collected from the six samples in the three groups was distinct (Fig. 3A, B). Further analysis of metabolites using a cluster heatmap showed that the abundance of lipid compounds in the model group was higher than that in the control group, while the abundance of nucleic acid compounds and carbohydrates in the model group was lower compared to the control group. G-Rd reduced the abundance of lipid compounds and increased the abundance of nucleic acid and carbohydrates compounds (Fig. 3C). A t-test and univariate analysis of metabolites showed that the content of linolelaidic acid (C18:2N6T) in the model group was significantly lower than that in the control group (*p* < 0.01). G-Rd treatment restored the content (*p* < 0.05 vs. model group, Fig. 3D). Correlation analysis (Fig. 3E) revealed that *F. rodentium* was positively correlated with TG (*p* < 0.001), TC (*p* < 0.001), ALT (*p* < 0.05) and AST (*p* < 0.001), whereas *M. intestinale* was positively corrected with linolelaidic acid (C18:2N6T) (*p* < 0.01), and negatively correlated with TG (*p* < 0.001), TC (*p* < 0.001), ALT (*p* < 0.05). In addition, 6,7-diketoLCA levels were significantly decreased in the model group but significantly increased in the G-Rd treatment group. The correlation analysis displayed that 6,7-diketoLCA was negatively correlated with *Faecalibaculum rodentium* and positively correlated with *Muribaculum intestinale*, suggesting that G-Rd may regulate gut microbiota to influence bile acid metabolism, thereby improving MAFLD.

Key protein targets in the liver were examined by Western blot analysis to further investigate the mechanisms through which G-Rd improves lipid metabolism. G-Rd normalised the protein expressions of p-PI3K (*p* < 0.01), p-AKT (*p* < 0.05) and SREBP-1c (*p* < 0.01) compared to those in the model group (Fig. 3F, G), suggesting that the mechanisms of G-Rd in improving lipid metabolism are at least partly related to PI3K/AKT/SREBP signaling pathway in MAFLD mice.

### G-Rd inhibited ferroptosis in hepatocytes of MAFLD mice

**Fig. 4:**
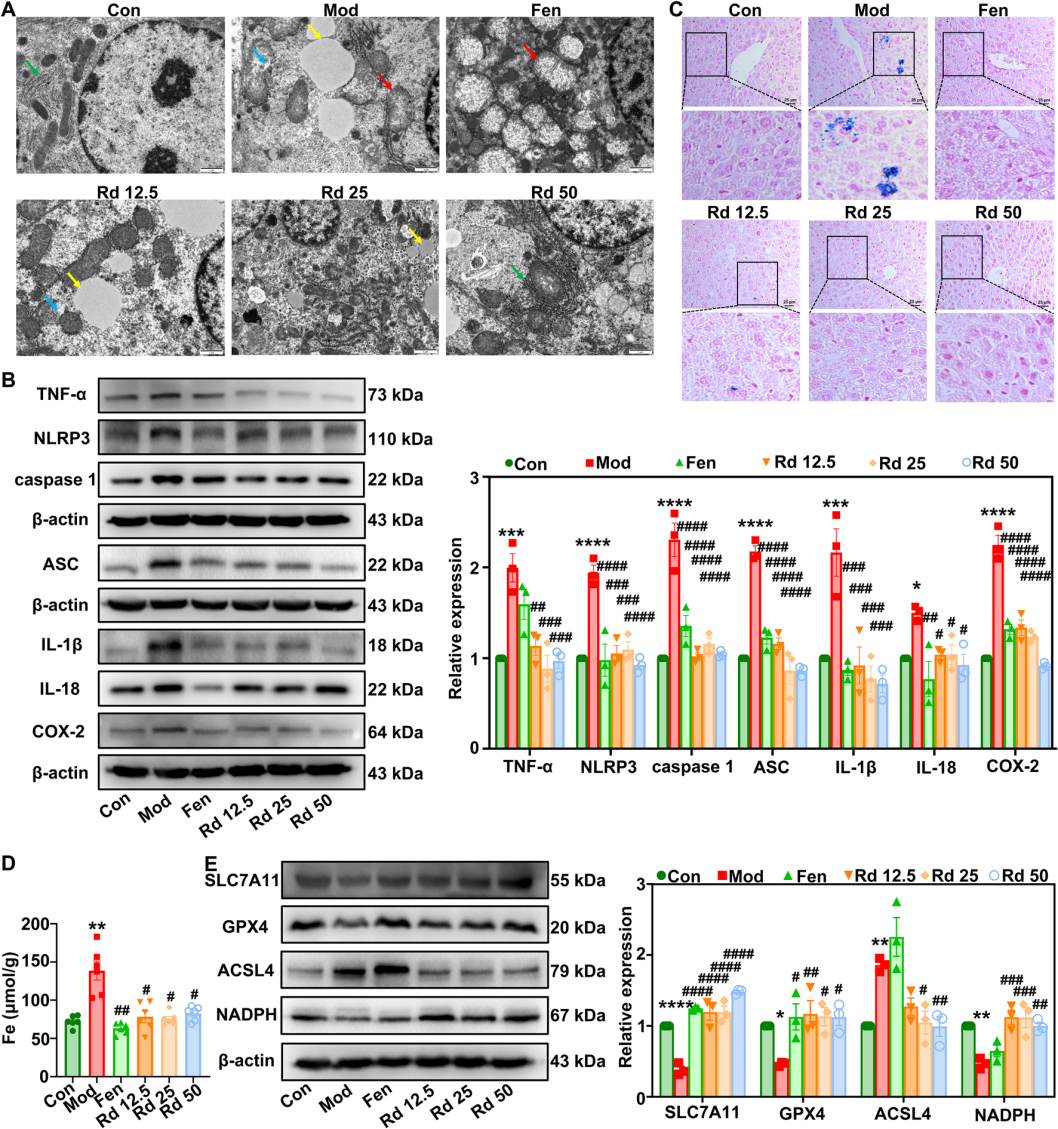
G-Rd exhibited anti-ferroptosis effects in MAFLD mice (n = 6 per group, ≥ 3 individual experiments each sample). **A** Representative images of hepatic ultrastructure, Scale bar = 1 μm. **B** Protein expressions and statistical analysis of TNF-α, NLRP3, cleaved caspase-1, ASC, IL-1β, IL-18, COX-2 normalized to β-actin. **C** Perl’s iron staining (scale bar = 25 μm. **D** Iron content. **E** Protein expressions and statistical analysis of SLC7A11, GPX4, ACSL4, NADPH to β-actin. *p < 0.05, **p < 0.01, ***p < 0.001, ****p < 0.0001 *vs.* Con; ^#^*p* < 0.05, ^##^*p* < 0.01, ^###^*p* < 0.001, ^####^*p* < 0.0001 *vs.* Mod group. Con: Control group, Mod: Model group, Fen: Fenofibrate group, Rd 12.5: 12.5 mg/kg ginsenoside Rd group, Rd 25: 25 mg/kg ginsenoside Rd group, Rd 50: 50 mg/kg ginsenoside Rd group

Ferroptosis is an important pathological event in lipid peroxidation, disrupting lipid metabolism in MAFLD. Since the primary functional role of G-Rd is associated with regulating lipid metabolism through the modulation of gut microbiota, detailed molecular events related to ferroptosis in hepatocytes were examined to investigate whether the action of G-Rd is associated with ferroptosis. The ultrastructure of the liver observed under TEM revealed mitochondrial swelling and rupture in the model group. Meanwhile, the rough endoplasmic reticulum was significantly reduced, along with several large lipid droplets with low electron density (polyunsaturated fatty acids), and single smaller lipid droplets with medium electron density, suggesting impaired oxidative metabolic capacity. In contrast, the G-Rd groups appeared to preserve normal mitochondria, exhibiting relatively intact morphology and mitochondria structure. They also displayed higher expressions of endoplasmic reticulum proteins and fewer lipid droplets (Fig. 4A). Additionally, G-Rd (12.5–50 mg/kg) dose-dependently reduced inflammatory protein expressions in the liver tissues, including TNF-α, NLRP3, cleaved caspase-1, ASC, IL-1β, IL-18 and COX-2, compared to the model group (Fig. 4B).

The potential effect of G-Rd in reducing ferroptosis was further explored by Perl’s iron staining and tissue iron content determination. Compared with the control group, both the positive expression of Perl’s iron staining (Fig. 4C) and iron content (Fig. 4D) in the liver of the model group were significantly increased (*p* < 0.01). Conversely, in the G-Rd group, both the positive expression of Perl’s iron staining and iron content were significantly decreased compared with the model group (*p* < 0.05). At the protein level, G-Rd treatment markedly restored the expression of ferroptosis-related proteins, including SLC7A11 (*p* < 0.0001), GPX4 (*p* < 0.05 or *p* < 0.01) and NADPH (*p* < 0.01 or *p* < 0.001), while reducing protein expression of ACSL4 (*p* < 0.01 or *p* < 0.001) compared to the model group (Fig. 4E), suggesting its potential anti-ferroptosis effects.

### G-Rd upregulated antioxidant defense and downregulated lipid peroxidation via the Nrf2 pathway

**Fig. 5:**
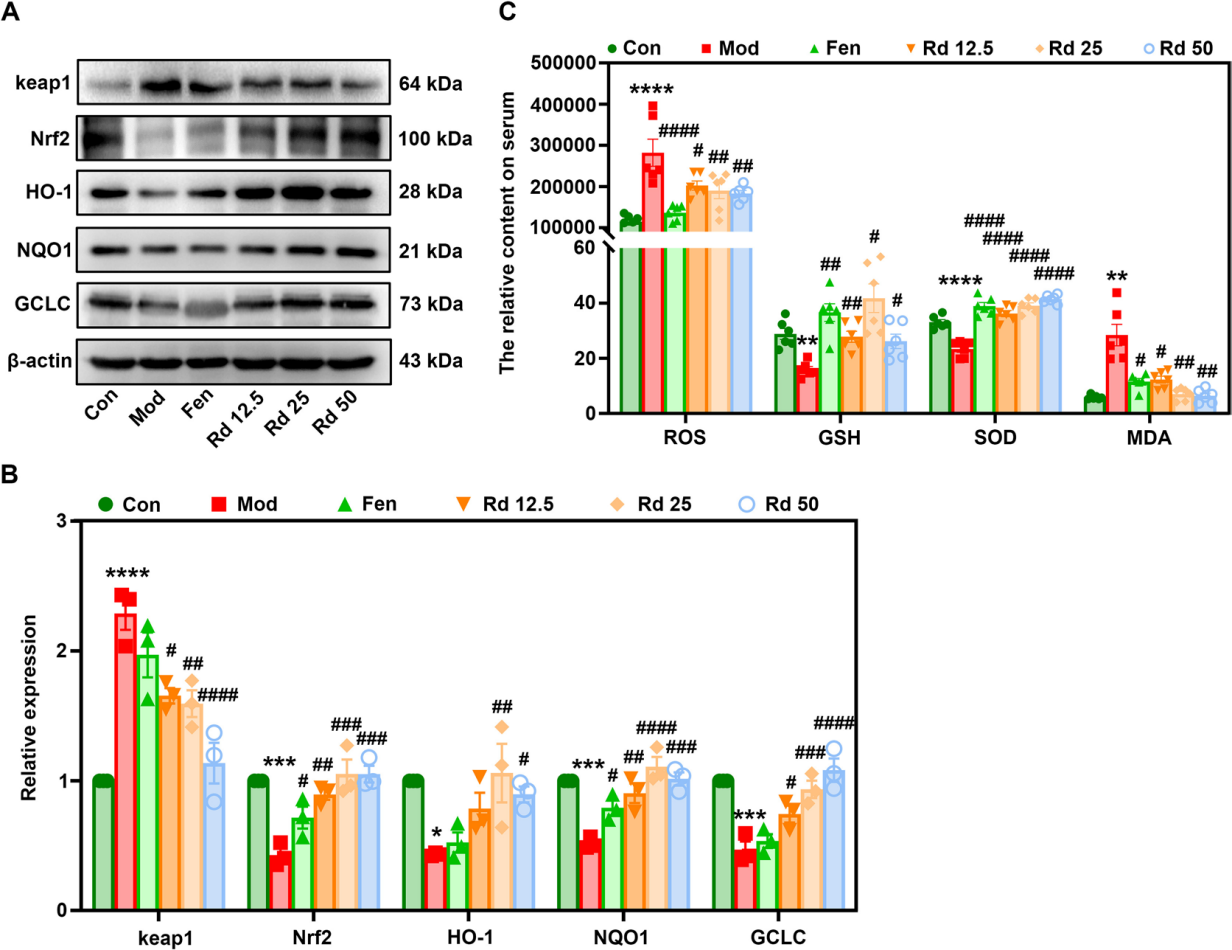
G-Rd exhibited antioxidant effects to inhibit lipid peroxidation via the Nrf2 pathway (n = 6 per group, ≥ 3 individual experiments per sample). **A** Immunoblotting analysis and **B** statistical analysis of relative protein expressions of keap1, Nrf2, HO-1, NQO1, GCLC normalized to β-actin. **C** Expressions of ROS, GSH, SOD and MDA. *p < 0.05, **p < 0.01, ***p < 0.001, ****p < 0.0001 *vs.* Con; ^#^*p* < 0.05, ^##^*p* < 0.01, ^###^*p* < 0.001, ^####^*p* < 0.0001 *vs.* Mod group. Con: Control group, Mod: Model group, Fen: Fenofibrate group, Rd 12.5: 12.5 mg/kg ginsenoside Rd group, Rd 25: 25 mg/kg ginsenoside Rd group, Rd 50: 50 mg/kg ginsenoside Rd group

The molecular mechanism underlying the anti-ferroptosis effect of G-Rd was further investigated, focusing on its antioxidant activity, particularly in relation to the Nrf2 signaling pathway, which acts as the master regulator of cellular antioxidant defense. Firstly, G-Rd treatment suppressed the protein expression of keap1 compared to the model group (Fig. 5A, B). Subsequently, G-Rd significantly restored the expression of Nrf2, as well as the downstream antioxidant proteins, including HO-1, NQO1, and GCLC, compared to the model group.

Furthermore, G-Rd significantly reduced ROS and MDA levels compared to the model group, while restoring antioxidant enzymes such as GSH and SOD. These findings highlight G-Rd’s powerful antioxidant activity through the upregulation of the keap1/Nrf2 signaling pathway (Fig. 5C).

### Role of gut microbiome disruption in the effect of G-Rd against MAFLD

The impact of gut microbiome disruption on MAFLD mice was examined by introducing antibiotics, with or without G-Rd treatment.

**Fig. 6:**
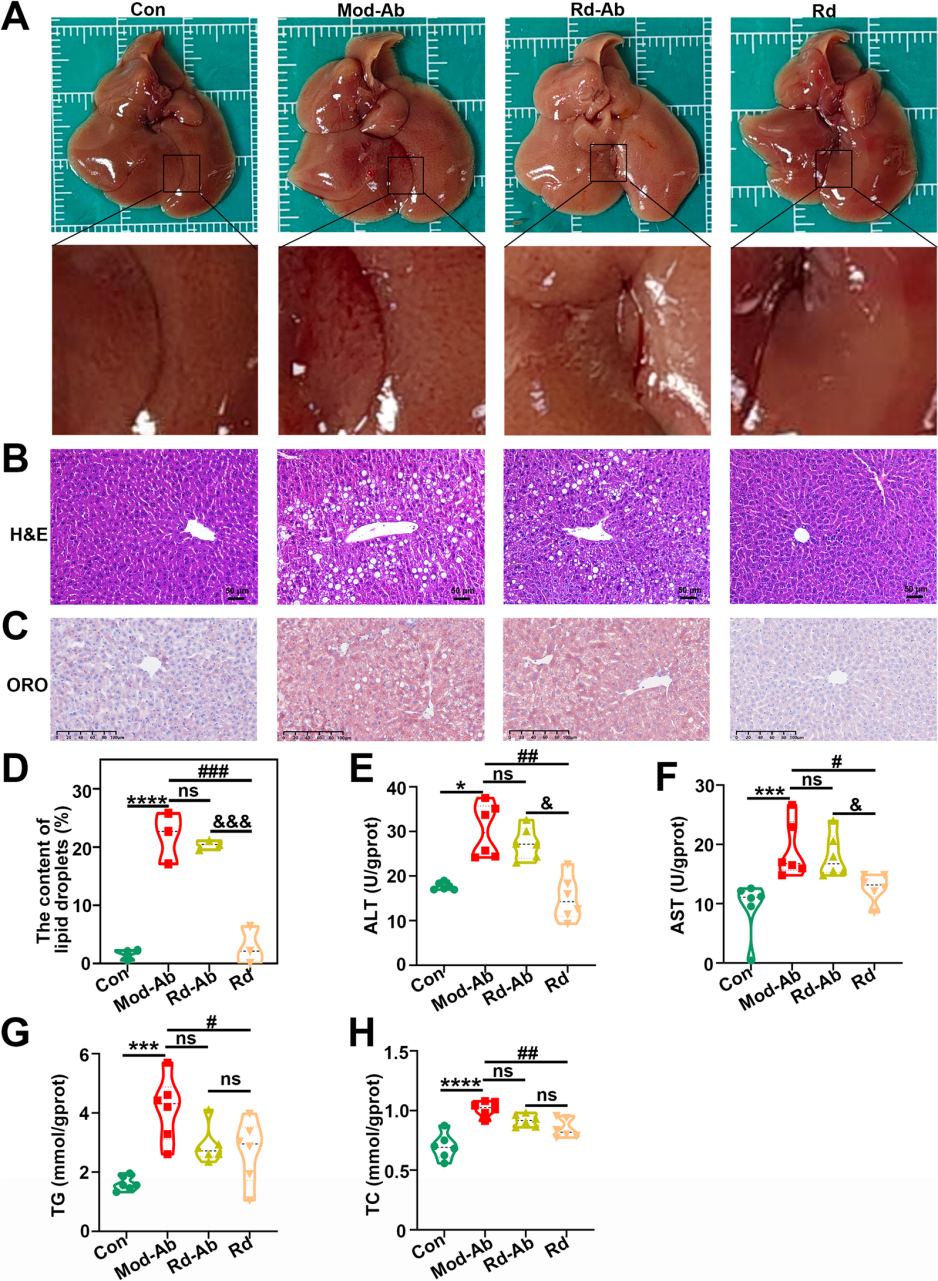
Role of antibiotics in gut microbiome disruption and the effect of Rd on MAFLD development. (n = 6 per group, ≥ 3 individual experiments per sample). **A** Liver appearance observed under a light microscope, **B** H&E staining (scale bar = 50 μm), **C** Oil Red O staining (scale bar = 100 μm). **D** Positive rate of Oil Red O staining (%), representing the lipid droplet content. Liver levels of ALT (**E**), AST (U/gropt) (**F**) and TG (**G**) and TC (**H**). *p < 0.05, ***p < 0.001, ****p < 0.0001 *vs.* Con; ^#^*p* < 0.05, ^##^*p* < 0.01, ^###^*p* < 0.001 *vs.* Mod group; ^&^*p* < 0.05, ^&&&^*p* < 0.001 *vs.* Rd. Con: Control group, Mod-Ab: antibiotic + HFD group, Rd-Ab: antibiotic + HFD + Rd group, Rd: HFD + Rd 25 mg/kg group

Visual inspections revealed that the liver tissue of the Mod-Ab group appeared pale, with some yellowish-white lipid droplets, while the G-Rd treatment and control groups appeared dark reddish and smooth, resembling normal liver tissue (Fig. 6A). Notably, the Rd-Ab group also appeared pale, similar to the model group. H&E staining results (Fig. 6B) indicated that both the Mod-Ab and Rd-Ab groups exhibited hepatocyte steatosis, ballooning degeneration and abnormal hepatocyte arrangement compared to the control and G-Rd groups. In the Oil Red O staining, the Rd group showed a significantly smaller number of neutral lipid droplets in the liver tissue compared to the Mod-Ab group (Fig. 6C and D), whereas the Rd-Ab failed to show such improvement. Additionally, Rd-Ab treatment did not attenuate liver injury or reduce elevated blood lipids in MAFLD mice, as evidenced by high levels of ALT, AST, TG and TC, which were comparable to those in the Mod-Ab group (Fig. 6E, F).

### Verification of G-Rd’s regulatory effect on gut microbiota in ameliorating MAFLD

**Fig. 7:**
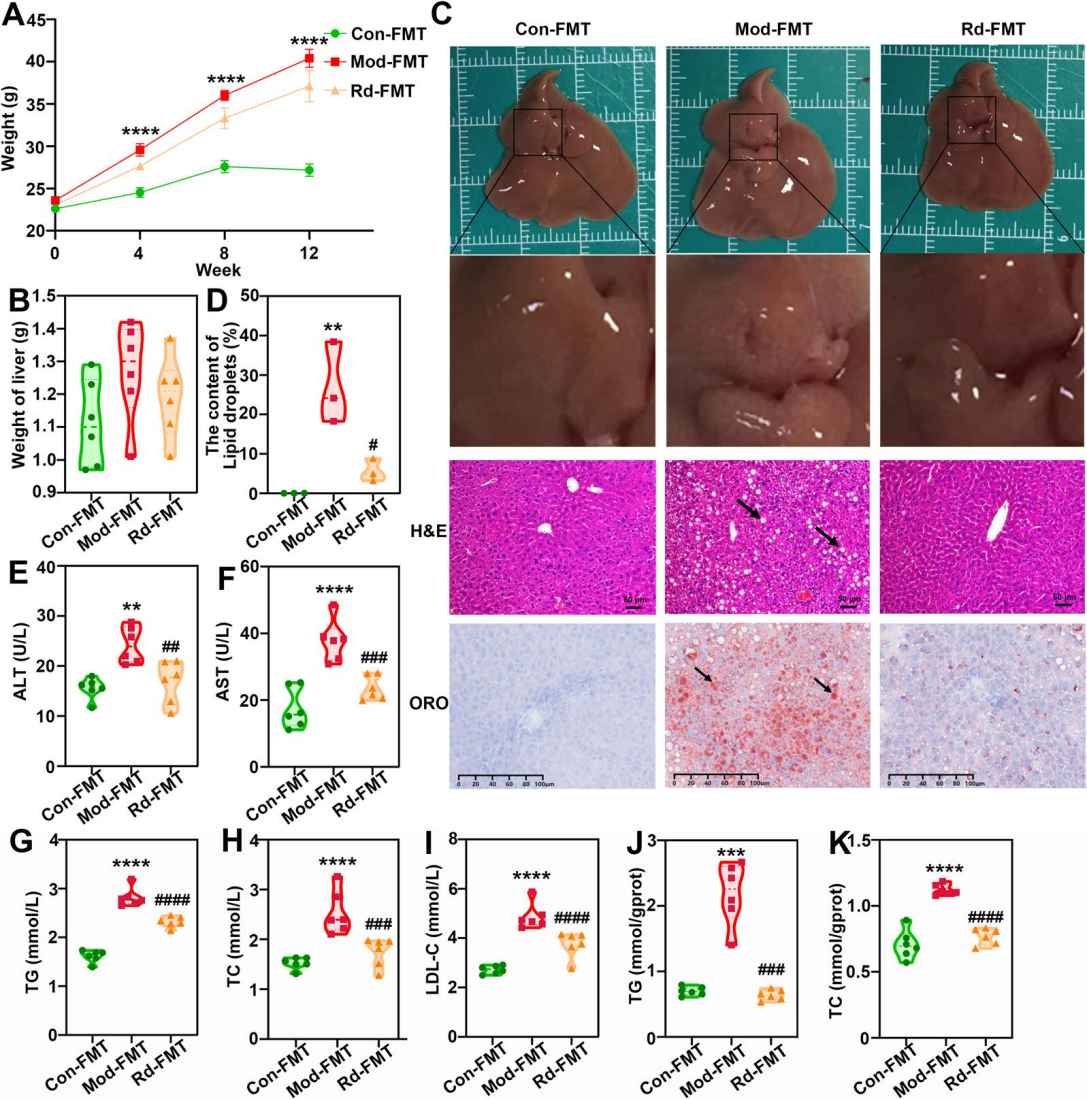
G-Rd alleviated liver injury and hepatic steatosis in a gut-dependent manner (n = 6 per group, ≥ 3 individual experiments per sample). **A** Body weight (g), **B** Liver weight (g). Representative **C** images of hepatic morphology, H&E staining (scale bar = 50 μm), and Oil Red O staining (scale bar = 100 μm). **D** Positive rate of Oil Red O staining (%), representing the lipid droplet content. Serum levels of ALT (**E**), AST (U/L) (**F**) and TG (**G**), TC (**H**) and LDL-C (**I**). Levels of TG (**J**) and TC (**K**). **p < 0.01, ****p < 0.0001 *vs.* Con; #*p* < 0.05, ^##^*p* < 0.01, ^###^*p* < 0.001, ^####^*p* < 0.0001 *vs.* Mod group. Con: Control-FMT group, Mod: Model-FMT group, Rd: 25 mg/kg ginsenoside Rd-FMT group

To investigate the role of the gut microbiota in G-Rd’s effects on MAFLD, an antibiotic cocktail was used to deplete the gut microbiota in HFD-fed mice, with or without G-Rd supplementation. After depleting the gut microbiota with the antibiotic cocktail, the body weight of the model-FMT group remained significantly lower than that of the control-FMT group. However, no significant difference in body weight was observed between the G-Rd-FMT and model groups (Fig. 7A). A similar trend was observed in liver weight changes (Fig. 7B).

Histopathological examination and Oil Red O staining showed fewer ballooned hepatocytes and a relative intact hepatic structure with uniform and orderly cell arrangement in the G-Rd-FMT group. In contrast, the model group exhibited significant lipid droplet accumulation and higher positive Oil Red O staining expression (Fig. 7C). Similarly, G-Rd-FMT treatment significantly reduced serum transaminase enzymes, including AST and ALT (Fig. 7E, F), as well as blood lipids (Fig. 7G–I), including TG, NEFA, TC and LDL-C, in addition to liver blood lipids (Fig. 7J, K), such as TG and TC, compared to the excessive levels observed in the model group. Taken together, these results suggested that an intact gut microbiota is essential for G-Rd’s amelioration of MAFLD.

**Fig. 8:**
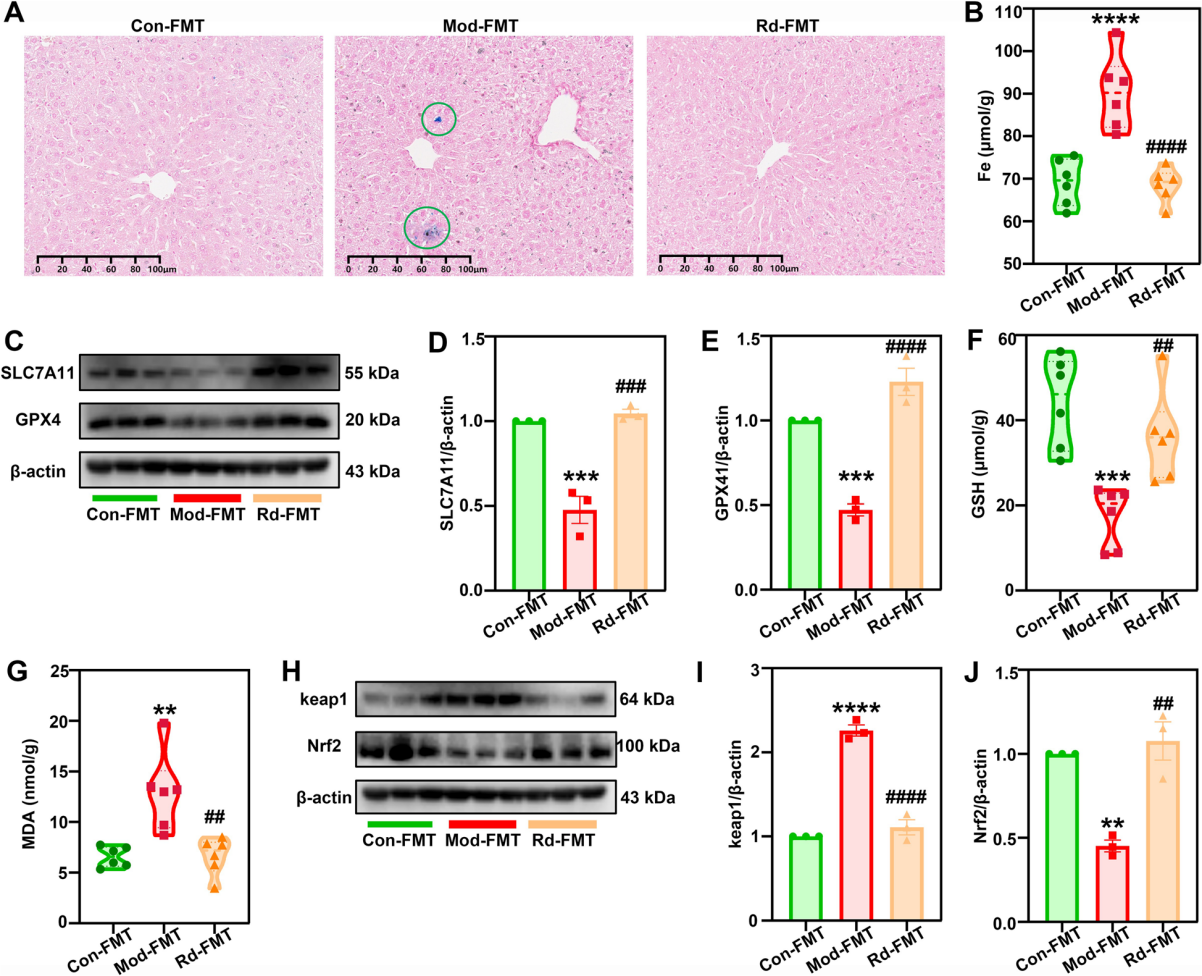
G-Rd exhibited anti-ferroptosis effects in a gut-dependent manner (n = 6 per group, ≥ 3 individual experiments each sample). **A** Perl’s iron staining (scale bar = 100 μm). **B** Iron content. **C** Immunoblotting analysis and (**D**, **E**) statistical analysis of relative protein expressions of SLC7A11, GPX4 normalised to β-actin. Expressions of GSH (**F**), and MDA (**G**). **H** Immunoblotting analysis and statistical analysis (**I, J**) of relative protein expressions of = Nrf2 and keap1 normalized to β-actin. **p < 0.01, ***p < 0.001, ****p < 0.0001 *vs.* Con; ^##^*p* < 0.01, ^###^*p* < 0.001, ^####^*p* < 0.0001 *vs.* Mod group. Con: Control-FMT group, Mod: Model-FMT group, Rd: 25 mg/kg ginsenoside Rd-FMT group

We further analyzed the effect of an intact gut microbiota on G-Rd-induced anti-ferroptosis in the liver tissues of mice with MAFLD. G-Rd-FMT significantly reduced the positive expression of Perl’s iron staining and the liver iron content (*p* < 0.0001) compared to the model group (Fig. 8A, B). Additionally, the G-Rd-FMT group upregulated the protein expressions of SLC7A11 and GPX4 (*p* < 0.001) compared to the model group (Fig. 8C–E). G-Rd-FMT also lowered the elevated lipid peroxide content of MDA and restored GSH levels compared to the model group (Fig. 8F, G). At the protein level, the G-Rd-FMT group significantly normalised key protein expressions in the Nrf2 signaling pathway, including Nrf2 and keap1 (Fig. 8H–J). These findings suggested that the Nrf2-mediated antioxidant capacity, coupled with the role of an intact gut microbiota, plays a crucial part in combating ferroptosis and lipid disruption occurred in MAFLD.

## Discussion

Currently, effective pharmacological treatments for the prevention and management of MAFLD are lacking. G-Rd, a key active ginsenoside found in *Panax* species, has demonstrated significant protective effects against hepatic injury in mice with HFD- and CCl_4_-induced liver diseases [23, 33]. The present study highlights the therapeutic potential of G-Rd in alleviating liver damage in an HFD-induced MAFLD mouse model and provides a detailed exploration if its mechanisms, particularly in regulating the gut microbiome-ferroptosis pathway. This research offers new insight into the complex mechanisms underlying ginsenoside Rd’s effect on MAFLD.

A 4-week treatment with G-Rd significantly reduced the levels of AST, ALT, TG and TC, which aligns with previous studies in HFD mice and CCl_4_-induced acute liver injury models [23, 24]. Notably, G-Rd also lowered RBG levels and alleviated MAFLD-related hypertrophy and hepatic steatosis. While Fen showed a considerable effect in reducing FBG, TG and TC levels, its ability to regulate AST and ALT levels was limited in this study. Fen is well established, FDA-approved treatment for hypertriglyceridemia and dyslipidemia, commonly used as a positive control in many MAFLD studies [34]. Previous research has shown that Fen improves liver function, reduces liver injury, and lowers serum TG and TC in HFD-induced MAFLD mice [35, 36]. However, a clinical study noted that after two weeks of Fen treatment, serum ALT and AST levels increased in patients [37]. Some in vitro studies have suggested that the elevated serum ALT and AST levels following Fen treatment are due to increased transaminase synthesis in hepatocytes rather than transaminase leakage caused by hepatocytes lysis [38].

Since there is a close link between HFD-induced gut microbiota dysbiosis and the increased risk of MAFLD, the underlying mechanisms of G-Rd in regulating the gut microbiota were explored in this study. First, G-Rd was found to regulate the overall diversity and abundance of the dominant taxa in the gut microbiome based on the microbiome profile post-trial. At the phylum level, G-Rd markedly reduce the level of *Firmicutes* and subsequently the ratio of *F/B*, a common marker used to assess overweight and obesity [39, 40]. At the genera level, G-Rd consistently reduced the excessively abundance of *Faecalibaculum* and *Limosilactobacillus*, both of which belong to the *Firmicutes* phylum, highlighting the importance of this phylum in the G-Rd-induced effects. *Faecalibaculum* has been recognized as a core gut microbes in various therapeutic agents, including Qijia Rougan decoction [41], lotus leaf (Nelumbo nucifera) [42], and Natto [43], in attenuating hepatic fibrosis and fatty liver accumulation in rats. At the species level, G-Rd significantly reversed the MAFLD-induced increase in *Faecalibaculum rodentium* and the decrease of *Muribaculum intestinale*. This result suggests that these two genera play a role in restoring gut microbiota homoeostasis under G-Rd treatment. *Faecalibaculum rodentium*, a bacterium within the *Firmicute* phylum, is predominant in obese mice [44] and is highly sensitive to alterations caused by a high-fat, high-cholesterol (HFHC) diet [45]. Furthermore, *F. rodentium* has been shown to have a strong correlation with hepatic lipids and is closely associated with γδ-T, CD1d, and lipid metabolites such as phosphatidylcholine (PC), phosphatidylethanolamine (PE), and phosphatidylglycerols (PGs). These findings reinforce previous results suggesting that the gut-microbiota regulates the homeostasis of hepatic γδ-T cells through lipid antigens such as PC, PE and PGs, which are recognized by CD1d [45, 46]. Moreover, the significant increase of *Bacillus faecalis* observed in mice on a HFD further decreased the levels of segmented filamentous bacteria [45], leading to a reduction in intestinal Th17 cells, promoting lipid absorption by intestinal epithelial cells, and contributing to obesity and metabolic syndrome [47, 48]. *Muribaculum intestinale*, a bacterium of the *Bacteroidota* phylum, is known to produce short-chain fatty acids such as propionate and plays a role in carbohydrate metabolism [49, 50]. Additionally, *Muribaculum intestinale* significantly influences T cell populations, contributing to immunoregulating of lipids [51]. The abundance of *Bacteroides intestinalis* is reduced in mice on a HFD [52]. As illustrated by Mcnamara et al. (2021), the abundance and diversity of beneficial bacteria in mice fed a high-sugar and HFD diet are notably diminished, including a reduction in *Muribaculum intestina* [49].

The functional analysis in this study revealed that lipid metabolism was a key factor in G-Rd’s regulatory effect on the gut microbiome. This corresponds to the finding that *Faecalibaculum rodentium* is involved in the modulation of G-Rd on the gut microbiome, and its functional role is related to lipid metabolism. Therefore, we conducted further analysis of the metabolism in MAFLD mice and found that G-Rd regulated lipid metabolism, effectively reversing the decrease in linolelaidic acid (C18:2N6T). These findings align with the significant reduction in linolelaidic acid (C18:2N6T) concentration observed in Chinese patients with hepatitis B-induced liver fibrosis and cirrhosis [53]. The molecular mechanism underlying the G-Rd’s effect is related to the regulation of the PI3K-AKT signaling pathway and the suppressed expression of SREBP-1c. SREBP-1c serves as a downstream protein of the PI3K/AKT/mTOR signaling pathway and plays a pivotal role in the lipogenic pathway. Key genes such as acetyl CoA carboxylase (ACC), ATP citrate lyase (acly), fatty acid synthase (FASN) and stearoyl COA desaturase (SCD1) are the main targets of SREBP-1c [54]. Mitigating SREBP-1c-mediated scd3 lipogenesis of monounsaturated fatty acids has been shown to effectively impede the occurrence of ferroptosis [55].

Ferroptosis plays a significant role in MAFLD and contributes to hepatocyte death through iron-dependent lipid peroxidation, exacerbating liver injury and disease progression. Exploration of the interplay between gut microbiota and lipid peroxidation offers a promising research strategy for MAFLD treatment and ferroptosis reversal. The molecular mechanism of G-Rd, based on the findings above, led to further investigation of its functional role in modulating ferroptosis in hepatocytes. In the present study, G-Rd ameliorated mitochondrial damage in the liver tissue of mice with fatty liver, increased GSH and SOD levels, and reduced lipid peroxide ROS and MDA levels. Altogether, the data demonstrated that G-Rd has the potential to reduce ferroptosis by rebalancing redox oxidant and lipid peroxidation, the key trigger of ferroptosis.

Nuclear factor E2-related factor 2 (Nrf2) is the major regulatory factor involved in protecting cells from oxidative stress. It can regulate redox balance through target genes such as SLC7A11 and GCLC/GCLM, thereby exerting an anti-ferroptosis function [56, 57]. Nrf2 can also enhance autophagy and inhibit ferroptosis through the Nrf2/HO-1 pathway [58]. Meanwhile, GPX4, a downstream enzyme of SLC7A11, is the only enzyme that can directly reduce complex phospholipid hydroperoxides. As a target gene of Nrf2, GPX4 helps prevent the rise in ROS levels and counteracts lipid peroxidation-induced ferroptosis [59, 60]. Our results demonstrated that the action of G-Rd was at least partly involved in Nrf2 activation, which attenuated ferroptosis and regulated lipid metabolism. To determine whether the action of G-Rd was predominantly mediated by Nrf2 or initiated by gut microbiome regulation, FMT-Rd was introduced in mice. Our results revealed that the regulatory of gut microbiome appeared to be the main action of G-Rd, triggering subsequent protection of hepatocytes by suppressing ferroptosis and lipid peroxidation. However, further research is needed to explore the crosstalk between Nrf2 activation and gut microbiota regulation, which may uncover novel therapeutic strategies for MAFLD. Additionally, exploring the potential synergy or interaction between Nrf2 activation and other cellular pathways involved in lipid metabolism and inflammation could provide a comprehensive understanding of G-Rd’s hepatoprotective effects. Further in vivo and in vitro studies could be performed to explore Rd’s effect on different types of liver cells and clarify the specific pathways involved. For instance, it should be determined whether G-Rd exhibits direct antioxidant effects on hepatocytes or if specific metabolites derived from the gut microbiota are mediating these effects in the liver. Moreover, how alterations in the diversity and abundances of certain species in the gut microbiota influence lipid metabolism and antioxidant pathways in the liver should be further explored. Although changes in gene and protein expression have been observed, no specific metabolites or signaling molecules have been pinpointed to explain these downstream effects. Additionally, the FMT experiments were inadequately designed, lacking analysis of the microbiota, metabolites, or the G-Rd content in the fecal solution used for transplantation.

## Conclusions

G-Rd mitigated HFD-induced MAFLD by reducing liver oxidative stress, lipid peroxidation, and ferroptosis through the modulation of the gut microbiota. The antioxidant and anti-ferroptotic effects of G-Rd, mediated by the Nrf2 pathway, played a key role in alleviating liver injury and hepatic steatosis in MAFLD. This study highlights the therapeutic potential of G-Rd for MAFLD and provides new insights into its mechanisms of action.

## Supplementary Information

Additional file1 (TIF 1051 KB)

Additional file2 (TIF 241 KB)

## Data Availability

All data supporting the conclusions of this article are included with this article.

## Abbreviations

G-Rd: Ginsenoside Rd
MAFLD: Metabolism-associated fatty liver disease
NASH: Non-alcoholic steatohepatitis
HFD: High-fat diet
ROS: Reactive oxygen species
MDA: Malondialdehyde
GSH: Glutathione
GPX4: Glutathione peroxidase 4
Fe^2+^: Ferrous ion
CCl_4_: Carbon tetrachloride
Fen: Fenofibrate
CMC: Carboxymethyl cellulose
H&E: Hematoxylin and eosin
RBG: Random blood glucose
AST: Aspartate transaminase
ALT: Alanine transaminase
TG: Triglycerides
TC: Total cholesterol
NEFA: Non-esterified fatty acids
LDL-C: Low-density lipoprotein cholesterol
PBS: Phosphate buffered saline
SOD: Superoxide dismutase
PCoA: Principal coordinate analysis
LDA: Linear discriminant analysis
LEfSe: Effect size
KEGG: Kyoto Encyclopedia of Genes and Genomes
UHPLC-MS/MS: Ultra-high performance liquid chromatography-tandem mass spectrometry
FA: Formic acid
CV: Coefficient of variation
NAS: MAFLD activity score
ORO: Oil Red O staining
TEM: Transmission electron microscopy
RIPA: Radioimmunoprecipitation
PCA: Principal component analysis
HFHC: High-cholesterol
PC: Phosphatidylcholine
PE: Phosphatidylethanolamine
PGs: Phosphatidylglycerols
ACC: Acetyl CoA carboxylase
Acly: ATP citrate lyase
FASN: Fatty acid synthase
SCD1: Stearoyl COA desaturase
Nrf2: Nuclear factor E2 related factor 2
