# Supplementary figures and images for: The gut microbiota-mediated ferroptosis pathway: a key mechanism of ginsenoside Rd against metabolism-associated fatty liver disease
Source: Chin Med. 2025 Jun 10;20:83. doi: 10.1186/s13020-025-01121-1 (PMC12150452; doi:10.1186/s13020-025-01121-1)

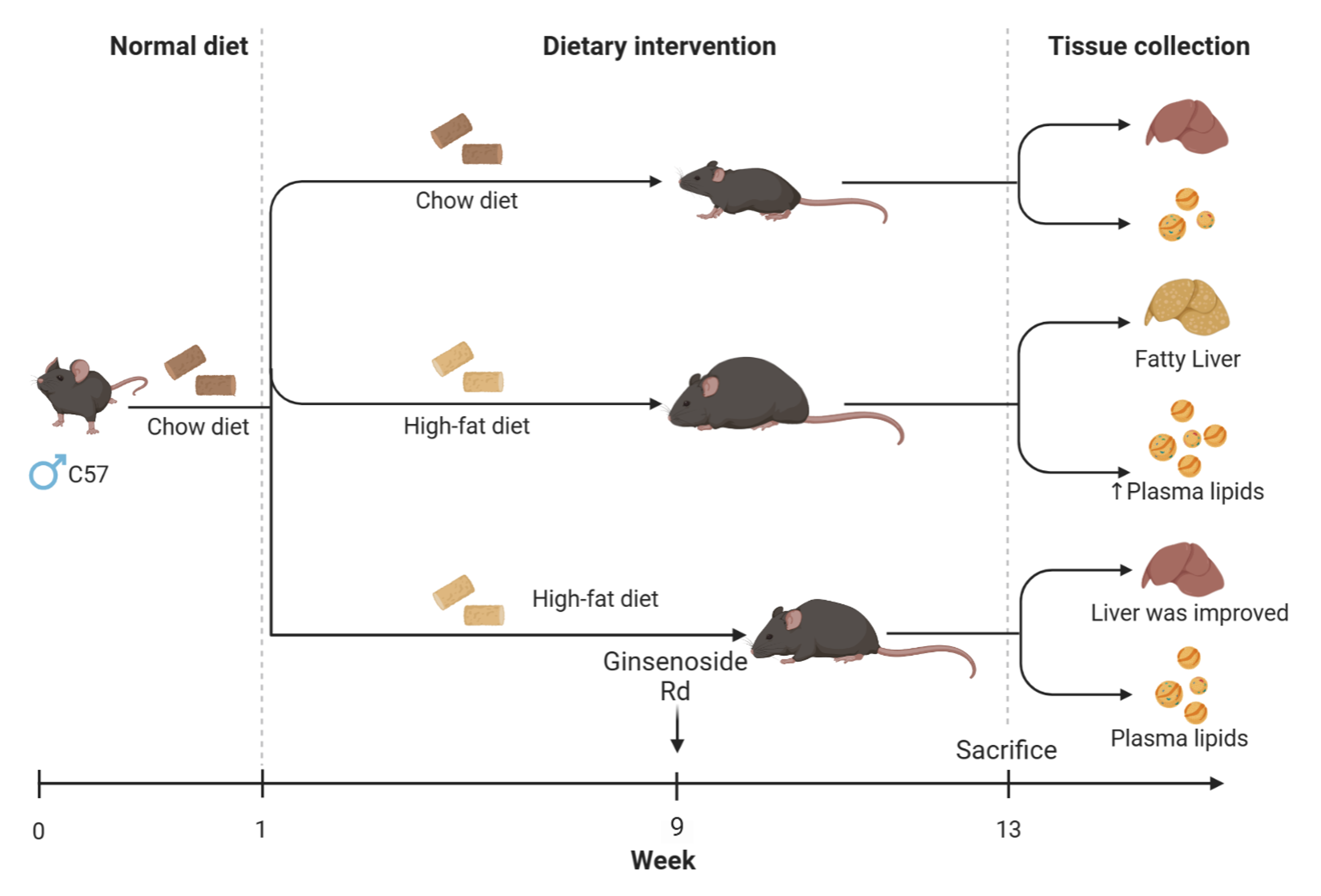

Supplement: Supplementary file 1 — Additional file1 (TIF 1051 KB) [file 13020_2025_1121_MOESM1_ESM.tif]

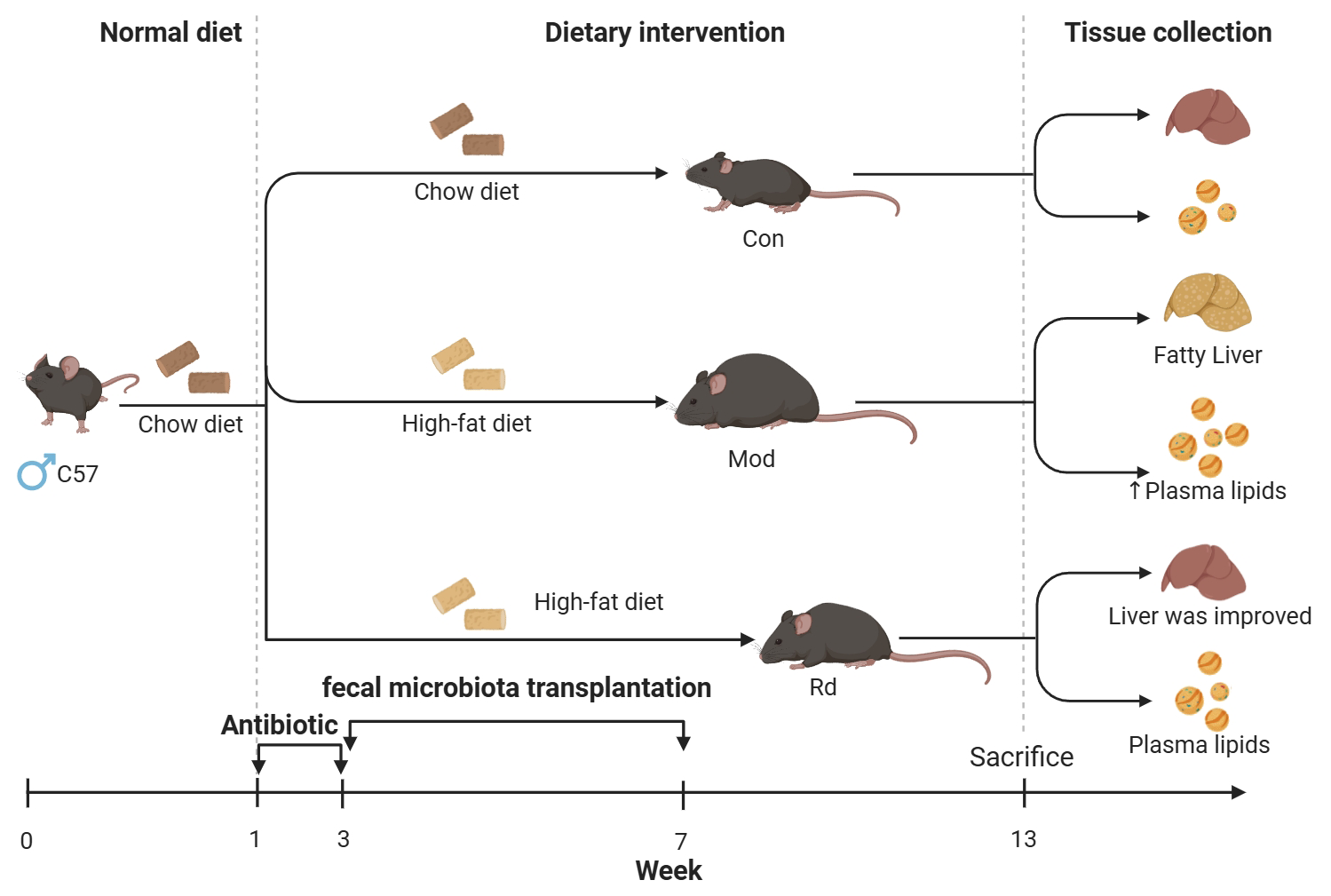

Supplement: Supplementary file 2 — Additional file2 (TIF 241 KB) [file 13020_2025_1121_MOESM2_ESM.tif]
